# Knowledge of COVID-19 and Its Influence on Mindfulness, Cognitive Emotion Regulation and Psychological Flexibility in the Indian Community

**DOI:** 10.3389/fpsyg.2020.589365

**Published:** 2020-11-12

**Authors:** Neha Dubey, Priyanka Podder, Dinkar Pandey

**Affiliations:** ^1^Department of Applied Psychology, University of Calcutta, Kolkata, India; ^2^Apollo Gleneagles Hospitals, Kolkata, India; ^3^Department of Psychology, University of Calcutta, Kolkata, India; ^4^Mental Health Foundation, Kolkata, India; ^5^Department of Computer Science and Technology (CST), IIEST Shibpur, Howrah, India

**Keywords:** knowledge of COVID-19, mindfulness, psychological flexibility, cognitive emotion regulation strategies, depression, anxiety, stress, India

## Abstract

The current global pandemic caused by COVID-19 has brought about an immense effect on the mental health of the general public. Considering the escalation in number of cases, mankind is facing a myriad of psychological problems, ranging from those related to taking precautions and maintaining safety to the ones caused by separation and bereavement. The current study aims to explore whether there is a significant difference between individuals with excellent, good, fair and vague knowledge of COVID-19 with respect to depression, anxiety, stress, level of mindfulness, specific cognitive emotion regulation strategies and psychological flexibility; to find out whether there is any significant relationship among these variables; and to determine whether knowledge of COVID-19, level of mindfulness, specific cognitive emotion regulation strategies and psychological flexibility are significant predictors of depression, anxiety and stress in the sample of the current study. The sample consisted of 402 individuals selected from the community following the research criteria. Data was collected using digital consent form, information schedule and questionnaires, from 3rd May to 13th May, 2020. The questionnaires consisted of a semi-structured interview schedule to assess knowledge of COVID-19, Depression, Anxiety and Stress Scale – 21, Five Facet Mindfulness Questionnaire-Short Form, Cognitive Emotion Regulation Questionnaire-Short version and Acceptance and Action Questionnaire-II. The data was statistically analyzed using analysis of variance test, correlational analysis and linear regression. The findings show that significant differences were present among individuals having varying degrees of knowledge of COVID-19 with respect to anxiety, level of mindfulness and psychological flexibility. Significant relationships were found to be present among the variables of the present study, having differing trends brought forward by the COVID-19 crisis. Certain socio-demographic characteristics and study variables were found to significantly predict the existing levels of depression, anxiety and stress in the current sample. The study suggests the necessity to formulate and implement appropriate mindfulness-based therapeutic interventions to address the mental health concerns arising as a result of the pandemic.

## Introduction

The coronavirus disease-19 (COVID-19) pandemic has put the world in a state of crisis and high alert. Basically, it is a novel ribonucleic acid coronavirus linked to the same family of viruses as severe acute respiratory syndrome (SARS). This mainly impacts the respiratory and digestive systems, the most common symptoms of which include fever, dry cough, difficulty breathing or shortness of breath, and tiredness, while the less common symptoms are body ache, sore throat, headache, loss of taste or smell, diarrhea, etc. It can take up to 2 weeks to show symptoms from the day one gets infected with the virus. The symptoms can range from mild to severe in nature and can be accompanied by septic shock and even systemic multiple organ failure. At the current state of knowledge, people in the elderly age group, in particular, are more susceptible to face death.

In order to deal with the COVID-19 pandemic, in the backdrop of escalating cases, on 22nd of March 2020, the Government of India enforced a 14-h voluntary public curfew. A nationwide lockdown was ordered soon after, from 24th of March 2020 for 21 days, strictly enforcing a number of regulations. Subsequently, considering the exponential rise in COVID-19 cases in India, the lockdown was extended in five more phases till June 2020.

The pandemic, the accompanying ever-increasing number of deaths, the lockdown, and the need for maintaining strict precautions against the virus can bring about an exponential rise in the mental health issues of the people affected by the pandemic as well (Kumar and Nayar, 2020; Li S. W. et al., 2020; Rehman et al., 2020; Verma and Mishra, 2020; Wang et al., 2020a; Zhu S. et al., 2020). According to Rajkumar (2020), a review of the existing literature on COVID-19 and mental health shows an increase in symptoms of depression, anxiety, self-reported stress, fear, and sleep disturbances. The concerns are more profound in children, older adults, and in those having pre-existing physical and mental health conditions as well as their caregivers.

The increase in psychological distress caused by the pandemic has also been reported by Bao et al. (2020). Substance abuse and social media exposure are also on the rise owing to prevailing situation (Rogers et al., 2020). Gao et al. (2020) found a high prevalence of mental health problems that is associated with frequent use of social media exposure in these times. Wang et al. (2020a) found that females, students, and those with specific physical symptoms like myalgia, dizziness, and coryza were significantly experiencing greater psychological impact of the pandemic by reporting higher levels of depression, anxiety, and self-reported stress. Similar findings have been reported among healthcare workers (Tan et al., 2020).

Various research has shown that psychosocial support is necessary to address the increasing mental health crisis. In order to create ample awareness about COVID-19, a number of initiatives were set up by the Government of India. Besides toll-free helpline numbers, several programs are being conducted across the offline and online platforms. Living with a novel disease like COVID-19 demands adequate scientific understanding of the same, including knowledge about the nature of the virus, necessary safeguarding guidelines and precautions to be taken, what to do if one gets sick, how to protect those who are most vulnerable, educate others regarding the virus, and prevent community spread. However, with India being a diverse and densely populated country, it is only fair to assume that not all sections of people shall receive and understand the mentioned guidelines in a uniform manner. Moreover, the chance of spreading several forwarded messages from unverified sources in social media platforms, focusing on myths rather than facts, impacts the people in various ways. It is obvious that the level of knowledge of COVID-19 can influence how one frames the reality of the “new normal,” be flexible, adapt and adjust with the changes posed by COVID-19, face the consequent mental health concerns, and employ adequate coping strategies to deal with the pandemic as well as be mindful of self and the environment.

Probing into the current level of mindfulness, the use of specific cognitive emotion regulation strategies and level of adaptability existing in a sample from the community can help to design appropriate intervention strategies to cope with the psychological impact of the pandemic. Psychological well-being has a high positive correlation with mindfulness; the latter mainly comprises awareness and non-judgmental acceptance of our moment-wise experience (Keng et al., 2011). This helps to avert distressing thoughts and emotions (Kabat-Zinn, 1990b; Hayes and Feldman, 2004). Baer (2003) defines mindfulness as “the non-judgmental observation of the ongoing stream of internal and external stimuli as they arise.” A similar definition of mindfulness has been given by Kabat-Zinn (1994).

Baer et al. (2004) proposed five aspects or facets of mindfulness that can help us in measuring this experience of being mindful. These are observation, description, mindful actions, non-judgmental inner experience, and non-reactivity to inner experience. Observation is basically the sensory awareness: the ways in which one sees, uses selective attention, and perceives and feels the internal and external world around the individual. Description entails the way of labeling one’s experiences and using words to express the same. Using mindful actions is the actions one undertakes after bringing a particular stimuli into focus and in the awareness of the present. It gauges whether one can get into the mode of making fast judgments while responding. Non-judgmental inner experience is the stance of accepting self and others as well as having unconditional empathy. This can enhance overall well-being and positivity to a great extent. The fifth aspect is that of having non-reactivity to inner experience. This is the ability to detach oneself from negative thoughts and emotions in order to facilitate acceptance of one’s existence and not be reactive to one’s experiences.

A substantial amount of research findings point to the fact that psychological well-being can be enhanced by the use of adaptive emotion regulation strategies (Extremera et al., 2019). According to those researchers, Garnefski and Kraaij (2007) posited that there are individual differences in the way different people self-monitor the effect of maladaptive emotions by using adaptive and maladaptive cognitive emotion regulation strategies. Garnefski et al. (2009) defined cognitive emotion regulation as the “conscious, mental strategies individuals use to cope with the intake of emotionally arousing information.” As per these researchers, cognitive emotion regulation involves four maladaptive and five adaptive strategies. The former constitutes rumination, self-blame, blaming others, and catastrophizing, while the latter comprises positive refocusing, refocusing on planning, acceptance, putting into perspective, and positive reappraisal.

Self-blaming entails blaming oneself for the negative experiences faced by an individual; other-blame refers to thoughts of blaming others or the environment for the negative experiences encountered by an individual; rumination occurs when an individual is preoccupied or thinks excessively about the feelings and thoughts associated with the negative event; catastrophizing entails the thoughts regarding explicitly emphasizing the terrible experiences an individual had to go through; putting into perspective is the strategy whereby one attempts to put less emphasis on the importance of the negative or stressful event; positive refocusing highlights engaging in thoughts regarding the positive aspects of one’s experiences instead of focusing on the actual negative or stressful event; positive reappraisal refers to thoughts of giving the event a positive meaning in terms of personal growth; acceptance is the strategy whereby one can accept to oneself regarding the actual happenings, while planning talks about deciding about the steps to be taken toward seeking solution and appropriately handling the negative or stressful event.

As per Extremera and Rey (2014), the four maladaptive cognitive emotion regulation strategies are associated with psychopathology, e.g., symptoms of anxiety, depression, anger, aggression, and distress. The other five adaptive cognitive emotion regulation strategies are related to positive mental health and well-being. Certain studies by Garnefski and Kraaij (2007); Aldao et al. (2010), and Fletcher et al. (2013) emphasized on similar findings.

Acceptance and psychological flexibility has been documented to have a positive effect on psychological well-being (Hayes and Strosahl, 2004). This construct is further understood by the principles underlying acceptance and commitment therapy by Hayes et al. (1999). According to Bond et al. (2006), psychological flexibility is defined as “contacting the present moment as a conscious human being, and, based on what that situation affords, acting in accordance with one’s chosen values” (Hayes et al., 2004). Bond et al. (2006) have mentioned that “psychological flexibility guides people in persisting with or changing their actions, in accordance with the values-based contingencies that they contact, when they are willing to experience the present moment.”

There are six key processes that are included in the process of psychological flexibility (Bond et al., 2006). The basic premise encompasses utilizing cognitive defusion and acceptance in order to experience the reality of the present moment and continue to transcend oneself and move toward a balanced lifestyle by committing actions which are in tune with one’s values. According to Hayes et al. (1999), cognitive defusion is the skill to be able to separate or distance one from the thoughts, let them come and go as they are, and noticing them instead of being caught up in them. It aims to separate thoughts from actions and create psychological distance between an individual and his/her thoughts. The second key factor, acceptance, according to Hayes (1994), is the process of “contacting the automatic stimulus functions of psychological events, without acting to alter (e.g., change, minimize, avoid) those functions.” Being in contact with the present moment is the third key process. “Defusion and acceptance help to foster such contact, and they are aided to this end by procedures that expand the range, sensitivity, depth, and purposive regulation of stimulus control processes so that people can better “attend” to broad or narrow ranges of stimulus events, as the current context demands.” The fourth aspect which deals with the contact with a transcendent version of oneself is necessary because “it is a perspective that is stable, and such stability and security can help people willingly to experience difficult cognitive content (e.g., fear). This stable sense of self can be experienced as “transcendent” or “spiritual,” because the limits of this deictic repertoire cannot be consciously contacted by the individual engaging in it” (Hayes et al., 1999; Barnes-Holmes et al., 2001). Hayes et al. (2004) defined values as “chosen qualities of action patterns (e.g., being a good manager and partner) that people can work toward, but that they cannot arrive at once-and-for-all (i.e., people have to work constantly at being a good worker and partner or they cease to be one). To the extent that people act according to their chosen values, they are living an effective life, for them.” This value-based living ensures that one engages in committed action in the future. All of these domains work together to enhance psychological flexibility and thus overall mental health of an individual.

In the context of COVID-19, the factors, as discussed above, can contribute to the extent of adaptive functioning of an individual and mental health issues associated with the pandemic. The level of knowledge of COVID-19 can influence how one chooses to act during the pandemic and influence one’s stance toward life during this difficult time. These in turn also influence the ability to be aware of one’s moment-to-moment experience, adapt, employ adaptive coping skills, have a non-judgmental approach, and look after one’s well-being.

## Materials and Methods

### Objectives

The objectives of the present study were as follows:

(1) To explore whether there is a significant difference between individuals with excellent, good, fair, and vague knowledge of COVID-19 with respect to depression, anxiety, stress, level of mindfulness, specific cognitive emotion regulation strategies, and psychological flexibility.
(2) To find out whether there is any significant relationship with respect to knowledge of COVID-19, depression, anxiety, stress, level of mindfulness, specific cognitive emotion regulation strategies, and psychological flexibility.
(3) To determine whether knowledge of COVID-19, level of mindfulness, specific cognitive emotion regulation strategies, and psychological flexibility are significant predictors of depression, anxiety, and stress in the sample of the current study.

### Sample

The study had a planned sample size of *n* = 385, the minimum sample size required to have margin of error within 5% at 95% confidence interval. Depression, anxiety, and stress were much prevalent among the Indian population during the lockdown, and Verma and Mishra (2020) found that 25, 28, and 11.6% of the sample of their study had moderate to extremely severe level of depression, anxiety, and stress. Our assumption was largely driven by the current uncertain social and economic environment around COVID-19, with mental well-being and psychological health likely to deteriorate. Specifically, the calculations were based on the formula recommended by the United Kingdom National Institute for Health Research Design Service, where *p* is the expected prevalence, *n* is the intended sample size, and the 95% confidence interval around the prevalence estimate is 1.96 × √(*p* × (1 − *p*)/*n*). Examining this *post hoc*, we achieved a sample of 402 and the prevalence (highest for anxiety) rate of 32%, giving us a 4.5% margin of error at 95% confidence interval.

The study has been carried out from 15th April to 15th May 2020. This period observed strict lockdown across India. The sampling of the current study was done in two stages. In the first stage, the researchers initially made a list of 260 individuals, known to them, from the general community. They were approached through telecommunication, and 243 of them verbally consented to take part in the study and help in recruiting participants. Using the snowball sampling method, a list of 860 willing individuals were prepared. From among them, in the second stage, 430 individuals were selected using the simple random sampling technique of fishbowl method where we chose only the even numbered individuals to constitute the sample of the present study. The selected candidates were approached through telecommunication and given the link to the digital consent form, information schedule, and questionnaires. Finally, 417 of them accessed the link and these individuals consisted of the sample for the present study. However, this figure includes 15 individuals who were unwilling to allow for their data to be used for further analysis. Thus, the final sample size for the analysis was *N* = 402.

The sample met the following inclusion and exclusion criteria.

Inclusion criteria:

(1) Age range was fixed between 18 and 64 years,
(2) Fluent ability in reading and understanding English,
(3) Consented to participate in the study,
(4) Minimum educational qualification of 10th grade.

Exclusion criteria:

(1) Inability to access the digital questionnaires of the present study.

### Sample Demographics

The sample characteristics are given under the following headings in Table 1.

**TABLE 1:** Socio-demographic distribution.

		% Distribution
**Gender**	Female	37.3%
	Male	62.7%
**Age Category**	18–21	18.66%
	22–29	43.28%
	30–39	28.11%
	40–49	5.47%
	50–59	2.99%
	60 or above	1.49%
**Employment Status**	Employed	64.4%
	Student	29.1%
	Unemployed	6.5%
**Educational qualification**	Class 10 pass	3.73%
	Class 12 pass	6.47%
	Undergraduate	14.68%
	Graduate	30.35%
	Postgraduate	32.84%
	Professional degree	11.94%
**Family monthly income**	10,000 or below	12.69%
	Upto 25000	13.18%
	Upto 50000	13.93%
	Upto 1 lakh	17.16%
	Less than 5 lakhs	16.92%
	5–10 lakhs	6.22%
	More than 10 lakhs	6.47%
	Chose not to respond	13.43%

#### Gender

Out of the total sample, 62.7% were males and 37.3% were females (Table 1).

#### Age

It was found that 18.66% of the sample lay in the age range of 18 to 21 years, and 43.28 and 28.11% of the sample fell in the age range of 22 to 29 and 30–39 years, respectively. These three groups comprised most of the distribution. Further, 5.47, 2.99, and 1.49% lay in the age range of 40 to 49, 50–59, and 60 years or above, respectively (Table 1).

#### Employment

There were 29.1% students, 64.4% were employed, while 6.5% were unemployed (Table 1).

#### Educational Qualification

With respect to this domain, it was found that 3.73 and 6.47% of the sample were educated till the 10th and 12th grade, respectively; 14.68% studied up to undergraduate degree. Graduates comprised 30.35% of the sample, while 32.84% were postgraduates; 11.94% completed professional degree (Table 1).

#### Average Monthly Income of Family

Of the sample, 12.69, 13.18, 13.93, 17.16, 16.92, 6.22, and 6.47% fell in the categories of Rs. 10,000 or below, up to Rs. 25,000, up to Rs. 50,000, up to 1 lakh rupees, up to 5 lakh rupees, 5–10 lakh rupees, and more than 10 lakh rupees, respectively. Out of the total sample, 13.43% of the sample did not respond to this item (Table 1).

### Measures Used

#### Consent Form

The participants were given a consent form, at the beginning of the study, which included the purpose of the present study, information about confidentiality, and voluntary participation along with statement that no risk was involved in participation. Details of the researchers were mentioned.

#### Information Schedule

Sociodemographic details including name/initials, age, pincode, gender, educational qualification, occupation, family type, residence type, people currently staying with, average monthly family income, and current health issues were collected.

#### Semistructured Interview Schedule to Assess Knowledge of COVID-19

This was prepared with the aim of estimating the level of awareness and knowledge of COVID-19 in the general community sample of the study. The items attempted to look into the participant’s knowledge of symptoms of COVID-19, myth about type of season that can help to reduce COVID-19 symptoms, the prescribed minimum distance to be maintained from others in public, whether lockdown restrictions were being strictly followed in the participant’s area, awareness of the various initiatives by the Government of India in order to tackle COVID-19, and lastly, knowledge about the different wearables that have been made compulsory for use in public places by the Government of India. Awareness and knowledge of COVID-19 was measured using a score on the following list of five questions:

(a) Choose any three core symptoms that can be linked to coronavirus.
(b) Which type of season helps to reduce coronavirus symptoms?
(c) What is the prescribed minimum distance to be maintained from others in public?
(d) Which of the following initiatives by the Government of India do you know about? (four initiatives from the Government of India included).
(e) What has been made compulsory for use in public places by the Government of India?

Correct responses for questions a, b, c, and e were given a score of 2, while knowledge of each initiative from the government carried a score of 1 giving question d a maximum score of 4.

The population was bucketed into 4 categories based on the overall scores achieved on the questionnaire:

(1) Excellent knowledge (scores > 10)
(2) Good knowledge (scores between 7 and 10)
(3) Fair knowledge (scores between 4 and 6)
(4) Incomplete/vague knowledge (scores < 4)

The distribution of the categories is shown below:

**Table:** 

Knowledge category	% of sample
Excellent knowledge	17.16%
Good knowledge	36.82%
Fair knowledge	22.89%
Incomplete/vague Idea	23.13%

#### Depression, Anxiety and Stress Scale-21 (DASS-21)

The Depression, Anxiety and Stress Scale-21 consists of three self-report subscales that can give measures of depression, anxiety, and stress for an individual over the past week. Each scale has seven items. This was prepared by Antony et al. (1998); the authors modified the original 42-item Depression, Anxiety and Stress Scale by Lovibond and Lovibond (1995) into this shorter 21-item version. In the current study, it has been used to assess subjective levels of depression, anxiety, and stress. Internal consistency reliability for depression, anxiety and stress scales was found to be 0.91, 0.80, and 0.84, respectively. The scoring was done in the form of a Likert scale from 0 to 3, where 0 stands for “never – did not apply to me at all,” 1 – “sometimes – applied to me to some degree, or some of the times,” 2 stands for “often, i.e., applied to me to a considerable degree or a good part of the time,” and 3 – “almost always – applied to me very much or most of the time.” The raw scores of all the three subscales were added separately and each multiplied by 2, thus arriving at three separate scores for all three subscales.

#### Five Facet Mindfulness Questionnaire-Short Form (FFMQ-SF)

This 15-item questionnaire was developed by Baer et al. (2008) from the original 39-item version of the Five Facet Mindfulness Questionnaire by Baer et al. (2006). This measures five aspects of mindfulness, namely, observation, description, mindful actions, non-judgmental inner experience, and non-reactivity to inner experience. This tool includes three items for each of the five facets. All of these provide an effective measure of self-awareness and mindfulness. According to Gu et al. (2016), the FFMQ-SF has high internal consistency. The factor structure of the short version is consistent with that of the long one. Large correlations exist between the total facet scores of both the versions, thus indicating that both versions measure highly similar constructs. Further, it was also found that both versions have similar high convergent validity. The scoring was based on a Likert scale from 1 to 5, where 1 stands for “never or very rarely true,” 2 signifies “rarely true,” 3 stands for “sometimes true,” 4 signifies “often true,” and 5 – “very often or always true.” The raw scores of all the five facets were added separately, considering the reverse scoring for certain items as mentioned by the authors. Then, scores of all five facets were added to get an overall score of mindfulness.

#### Cognitive Emotion Regulation Questionnaire-Short Version (CERQ-Short)

This short 18-item version of the Cognitive Emotion Regulation Questionnaire was developed by Garnefski and Kraaij (2006) from the 36-item version of the same questionnaire by Garnefski et al. (2001). As the tool is not freely available for use in research, permission was taken from the authors before including this tool in the study. The questionnaire has nine different conceptual subscales–self-blame, other-blame, rumination, catastrophizing, positive refocusing, planning, positive reappraisal, putting into perspective, and acceptance. These are grouped into two categories of negative and positive cognitive emotion regulation strategies. The former is comprised of the first four, while the latter consists of the last five strategies. There are two items in each subscale. In the present study, this tool has been used to measure the cognitive strategies that determine an individual’s pattern of responding to stressful events or situations. According to Garnefski and Kraaij (2006), the alpha reliability of CERQ-short subscales mostly ranged from 0.73 to 0.81. The validity of the tool has been found to be acceptably high. The scoring of this questionnaire is also based on a Likert scale, from 1 to 5, where 1 stands for “(almost) never,” 2 signifies “sometimes,” 3 stands for “regularly,” and 4 and 5 signify “often” and “(almost) always,” respectively. The scores of all nine different conceptual subscales were calculated separately and used in the analysis of the findings.

#### Acceptance and Action Questionnaire-II (AAQ-II)

This tool was developed by Bond et al. (2011). According to Hayes et al. (2004), this was developed to measure acceptance of private experiences or experiential avoidance, i.e., psychological flexibility. It consists of 10 self-report items and it shows good internal consistency (Cronbach’s α > 0.81). Further, this measure shows negative correlation with other tools of depression and anxiety like the Beck Depression Inventory-II (Beck et al., 1996; *r* = −0.75) and Beck Anxiety Inventory (Beck and Steer, 1993; *r* = −0.59). The scoring was done in the form of a Likert scale from 1 to 7, where 1 stands for “never true,” 2 – “very seldom true,” 3 stands for “seldom true,” 4 signifies “sometimes true,” 5 stands for “frequently true,” 6 – “almost always true,” and 7 – “always.” The scores of all the 10 items were added to arrive at the overall psychological flexibility score.

### Procedure

Data collection was conducted using digital consent form, information schedule, and questionnaires, from 3rd May to 13th May 2020. The format was prepared such that the participants needed to give consent in order to proceed with the study, the failure of which would lead to termination of participation. A particular sequence of administration of questionnaires was followed, and standardized instructions were provided for each of the questionnaires. There was an average completion time of 17 min. The sample of the present study was given the link to the digital consent form, information schedule, and questionnaires as well as the contact information of the researchers so that the latter can attend to any queries of the former. The questionnaires were presented in the following order: semistructured interview schedule to assess knowledge of COVID-19; Depression, Anxiety and Stress Scale-21; Five Facet Mindfulness Questionnaire-Short Form; Cognitive Emotion Regulation Questionnaire-Short Version; and Acceptance and Action Questionnaire-II. The responses of all those who accessed the link and gave data were saved in a structured format. The responses were then analyzed and interpreted.

### Analysis of Data

Data obtained from the tests was scored and tabulated. Statistical analysis of data was done with the help of Statistical Package for Social Sciences, IBM Windows Version 20 (SPSS 20.0) and R version 4.0.2.

**FIGURE 1:**
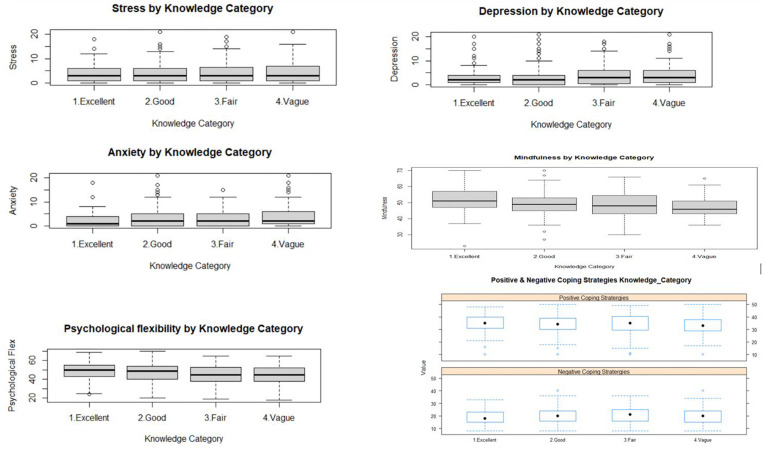
Findings in ANOVA variation shown in box plots.

In order to analyze the differences between groups with different levels of knowledge about COVID-19, the data was subjected to an analysis of variance (ANOVA) test. The test was used to ascertain group difference across various parameters like depression, anxiety, stress, level of mindfulness, specific cognitive emotion regulation strategies, and psychological flexibility. Anxiety, mindfulness, psychological flexibility, and putting into perspective were statistically significant (at 95% confidence level) (Table 2). The box plots depicting the variation across the knowledge category are shown in Figure 1.

**TABLE 2:** Showing the results obtained from Analysis of variance (ANOVA) comparing findings across different Knowledge of COVID-19 categories (excellent, good, fair, and vague).

Parameter	Df	Sum Sq	Mean Sq	*F* value	Level of significance
Stress	3	87	28.88	1.294	0.276
Depression	3	125	41.77	1.781	0.15
Anxiety	3	199	66.40	3.972	0.00827**
Mindfulness	3	783	261.12	5.117	0.00175**
Psychological flexibility	3	1118	372.8	3.596	0.0137*
Self-blame	3	3.3	1.103	0.210	0.889
Other-blame	3	18.9	6.294	1.491	0.216
Rumination	3	8.0	2.670	0.656	0.579
Catastrophizing	3	31.1	10.368	2.028	0.109
Positive refocusing	3	3.1	1.018	0.228	0.877
Planning	3	26.1	8.712	1.755	0.155
Positive reappraisal	3	1.2	0.412	0.089	0.9663
Putting into perspective	3	40.3	13.417	2.817	0.0389*
Acceptance	3	1.7	0.583	0.109	0.955

Significance codes: “**” 0.01 “*” 0.05.

**FIGURE 2:**
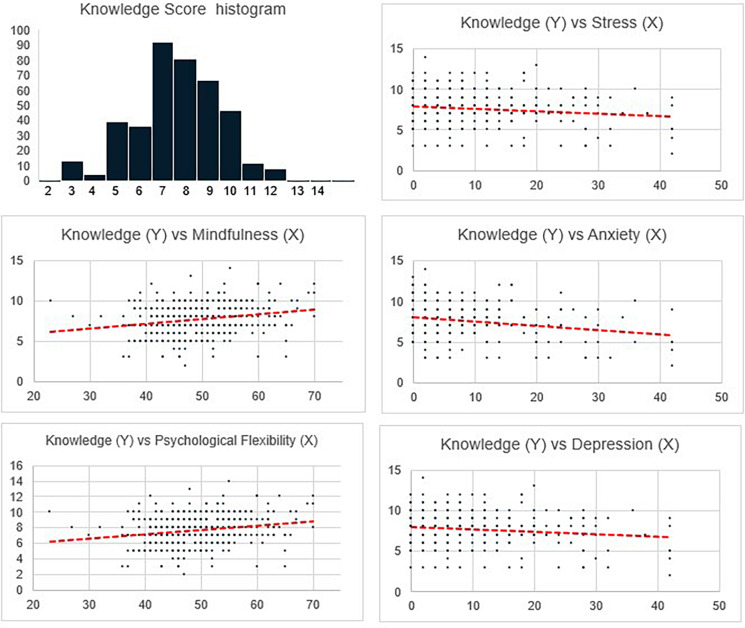
Correlation between stress, anxiety, depression, mindfulness and psychological flexibility with knowledge of COVID-19.

**FIGURE 3:**
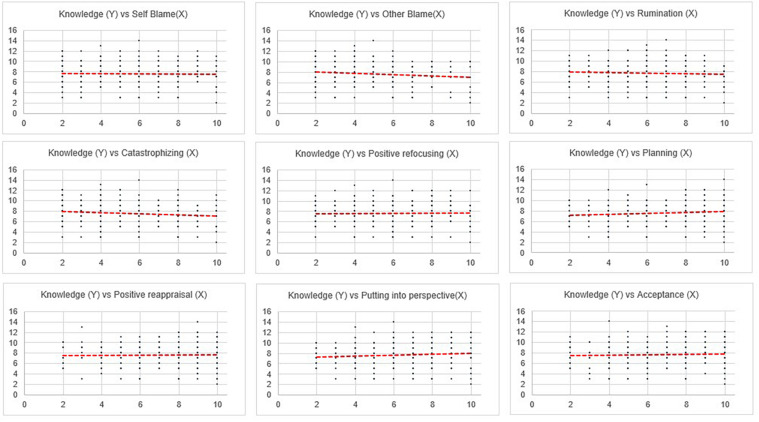
Correlation between specific coping strategies and knowledge of COVID-19.

To study the strength of relationship among the various parameters (as laid out in objective 2), correlation analysis was conducted (results in Table 3). With respect to the second objective, correlational analyses was computed between the variables (Table 3). The findings for correlation of knowledge of COVID-19 with the broad domains, namely, stress, anxiety, depression, overall mindfulness and psychological flexibility, were significant at the 0.01 level. The detailed correlational computation among the subdomains was further done. The findings of the same are shown in Table 4 and Figures 2, 3.

**TABLE 3:** Correlation results with Knowledge category (significant findings are highlighted in bold).

Correlation With Knowledge of COVID-19			
	Measured parameter	Co-efficient	Significance level (P)
**Broad domains**	Stress	−0.14	<0.01
	Anxiety	−0.21	<0.01
	Depression	−0.17	<0.01
	Overall mindfulness	0.21	<0.01
	Psychological flexibility	0.19	<0.01
**Coping strategies**	Self-blame	−0.02	0.64
	**Other-blame**	−**0.12**	**0.02**
	Rumination	−0.05	0.28
	**Catastrophizing**	−**0.13**	**0.01**
	Positive refocusing	0.01	0.84
	**Planning**	**0.11**	**0.03**
	Positive reappraisal	0.02	0.72
	Putting into perspective	0.09	0.08
	Acceptance	0.04	0.43

**TABLE 4:** Correlation among all the factors.

	Stress raw score	Anxiety raw score	Depre- ssion raw score	Obser- ving items	Descr- ibing items	Acting with awar- eness items	Non- judging items	Non- react- ivity items	Over- all mindfu- lness	Self- blame	Other- blame	Ruminat- ion	Catastro- phizing	Posit- ive refocu- sing	Plann- ing	Posit- ive reappr- aisal	Putt- ing into persp- ective	Accep- tance	Psychol- ogical flexib- ility	Know- ledge Score
Stress Raw Score	1																			
Anxiety Raw Score	0.754**	1																		
Depression Raw Score	0.831**	0.79**	1																	
Observing Items	0.203**	0.188**	0.153**	1																
Describing items	−0.098	−0.088	−0.116*	0.291**	1															
Acting with awareness items	−0.545**	−0.511**	−0.554**	−0.242**	0.168**	1														
Non-judging items	−0.496**	−0.472**	−0.506**	−0.337**	0.188**	0.589**	1													
Non-reactivity items	0.246**	0.217**	0.269**	0.536**	0.224**	−0.325**	−0.44**	1												
Overall Mindfulness	−0.274**	−0.265**	−0.302**	0.514**	0.728**	0.468**	0.405**	0.395**	1											
Self-blame	0.229**	0.238**	0.283**	0.333**	0.051	−0.329**	−0.379**	0.349**	0.012	1										
Other-blame	0.278**	0.309**	0.306**	0.171**	−0.026	−0.379**	−0.306**	0.226**	−0.124*	0.255**	1									
Rumination	0.309**	0.26**	0.304**	0.365**	0.086	−0.338**	−0.319**	0.391**	0.077	0.52**	0.346**	1								
Catastro- phizing	0.352**	0.337**	0.38**	0.246**	−0.109*	−0.408**	−0.408**	0.2**	−0.187**	0.432**	0.509**	0.524**	1							
Positive refocusing	0.112*	0.138**	0.081	0.339**	0.184**	−0.183**	−0.213**	0.343**	0.188**	0.264**	0.334**	0.36**	0.256**	1						
Planning	0.083	0.015	0.026	0.356**	0.239**	−0.070	−0.142**	0.326**	0.283**	0.38**	0.135**	0.403**	0.205**	0.378**	1					
Positive reappraisal	0.042	−0.035	0.002	0.342**	0.258**	−0.069	−0.113*	0.344**	0.304**	0.271**	0.12*	0.419**	0.222**	0.473**	0.565**	1				
Putting into perspective	0.073	0.007	0.111*	0.314**	0.154**	−0.217**	−0.193**	0.312**	0.149**	0.486**	0.236**	0.413**	0.316**	0.427**	0.51**	0.466**	1			
Acceptance	0.148**	0.082	0.19**	0.262**	0.191**	−0.157**	−0.173**	0.33**	0.179**	0.301**	0.161**	0.539**	0.318**	0.333**	0.405**	0.51**	0.363**	1		
Psycho- logical flexibility	−0.396**	−0.415**	−0.444**	−0.089	0.331**	0.469**	0.526**	−0.134**	0.437**	−0.295**	−0.321**	−0.315**	−0.543**	−0.062	0.041	0.018	−0.102*	−0.099*	1	
Knowledge Score	−0.144**	−0.215**	−0.169**	0.040	0.206**	0.143**	0.065	0.096	0.214**	−0.024	−0.118*	−0.054	−0.135**	0.010	0.109*	0.018	0.088	0.040	0.192**	1

Significance codes: “**” 0.01 “*” 0.05.

Finally, linear regression was used to analyze whether knowledge about COVID-19 and psychological/emotional coping strategies could predict the depression, anxiety, or stress level of an individual. The sample was divided into 80% training set and 20% hold out. A total of 19 different variables were used to predict anxiety level (Tables 5–7). These constituted eight demographic variables, namely, age, gender, no of years of education, employment status, living in nuclear family, urban flag, no. of people living with, and chronic condition flag. Eleven other behavioral factors were considered for the analysis, which included specific cognitive emotion regulation strategy, level of mindfulness, and psychological flexibility. The same set of 19 different predictors were used in order to analyze depression (Tables 8, 9) and stress (Tables 10, 11).

**TABLE 5:** Table showing findings of anxiety prediction, the model had an adjusted R-square of 25%.

Regression statistics	
Multiple R	0.525464379
R Square	0.276112814
Adjusted R Square	0.253782104

**TABLE 6:** The final model of anxiety regression with significant variables.

	Coefficients	Standard error	*t* Stat	*P*-value
Intercept	10.54	2.13	4.95	0.00
Gender 1=F 2=M	0.84	0.38	2.19	0.03
Self-blame	0.26	0.10	2.73	0.01
Overall mindfulness	−0.06	0.03	−1.98	0.05
Other blame	0.29	0.10	2.90	0.00
Positive refocusing	0.23	0.10	2.35	0.02
Putting into perspective	−0.26	0.10	−2.53	0.01
Psychological flexibility	−0.10	0.02	−4.76	0.00
Knowledge score	−0.21	0.10	−2.20	0.03

**TABLE 7:** Average scores of anxiety (gender-wise).

Gender	Average anxiety score
Female	3.0
Male	3.5
**Grand total**	**3.3**

**TABLE 8:** Table showing findings of depression prediction, the model had an adjusted R-square of 28%.

Regression statistics	
Multiple R	0.553525912
R Square	0.306390935
Adjusted R Square	0.281299134

**TABLE 9:** The final model of depression regression with the significant variables.

	*Coefficients*	*Standard Error*	*t Stat*	*P-value*
Intercept	10.93	2.47	4.43	0.00
Chronic Condition Flag	1.37	0.60	2.29	0.02
Self-blame	0.26	0.10	2.56	0.01
Overall Mindfulness	−0.12	0.03	−3.44	0.00
Other-blame	0.31	0.11	2.81	0.01
Acceptance	0.28	0.10	2.84	0.00
Psychological Flexibility	−0.12	0.03	−4.85	0.00

**TABLE 10:** Table showing findings of stress prediction, the model had an adjusted R-square of 22%.

Regression Statistics	
Multiple R	0.49409
R Square	0.244125
Adjusted R Square	0.220808

**TABLE 11:** The final model of stress regression with the significant variables.

	*Coefficients*	*Standard Error*	*t Stat*	*P-value*
Intercept	12.1	2.4	5.09	0.000
Chronic condition flag	1.5	0.6	2.54	0.011
Rumination	0.6	0.1	4.78	0.000
Overall mindfulness	−0.1	0.0	−3.4	0.001
Psychological flexibility	−0.1	0.0	−4.5	0.000

## Results

## Discussion

The coronavirus disease 2019 (COVID-19) pandemic has caused much stress and uncertainty in the current times (McCloy, 2020). Both in adults and children, fear about the novel virus, ensuing anxiety, being used to the “new normal” with regard to living everyday life, maintaining safety measures, and not knowing what the future beholds are common causes of immense distress (Alkwiese Sr et al., 2020; Cao et al., 2020; Ebrahim et al., 2020; Li S. W. et al., 2020; Moccia et al., 2020; Wang et al., 2020a; Zhou et al., 2020; Zhu S. et al., 2020; Özdin and Bayrak Özdin, 2020). Social distancing is often the cause of feeling isolated, lonely, and sad (Fegert et al., 2020; Hoffart et al., 2020; Loades et al., 2020; Moghanibashi-Mansourieh, 2020). As the news keep pouring in regarding the number of people infected, those undergoing treatment, those ultimately facing fatal outcomes, the staggering status of public health service, the unfateful plight of the poor, rising unemployment, and the grim situation faced by frontline workers (Kang et al., 2020; Lai et al., 2020; Pappa et al., 2020; Preti et al., 2020), police personnel, and emergency service providers, the feeling of being overwhelmed is on the rise (Luo et al., 2020).

Besides, those with various physical and psychological disabilities, physical and mental health conditions, staying at various kinds of homes (old age homes, those homes providing shelter and rehabilitation to children), residing alone far away from family owing to the lockdown or job requirements and families of frontline workers who are likely to get infected owing to greater exposure to the virus are specifically more vulnerable to mental health issues during these trying times (Liem et al., 2020; Rajkumar, 2020; Rashidi Fakari and Simbar, 2020; Tsai and Wilson, 2020; Yang et al., 2020; Yao et al., 2020; Zhu Y. et al., 2020).

We used ANOVA (analysis of variance) to explore the first objective of the study, i.e., to examine whether there is a statistically significant difference between knowledge of COVID-19 and the mentioned parameters. The findings (Table 2) show that there are significant differences between individuals with excellent, good, fair, and vague knowledge of COVID-19 with respect to anxiety, level of mindfulness, and psychological flexibility. However, there was no significant difference across groups with respect to stress and depression (variation across the knowledge category in Figure 1).

The governmental bodies in India are carrying out various awareness programs *via* online and offline media, accredited social health activists, non-governmental organizations, and the police, who are working in close collaboration to spread word about the nature of the virus, the means of safeguarding oneself from getting infected, guidelines to follow if one gets sick, ways to protect the vulnerable groups like those above 65 years of age and young children, and how to educate others regarding the virus and prevent community spread. They have repeatedly highlighted the need to wash hands; cover mouth while in public, when exposed to a patient, or while coughing and sneezing; wear masks or other face shields properly; maintain social distance; avoid crowds or gatherings; stay at home; and call up at given hotline numbers in case there is any emergency or need to report (Ministry of Health and Family Welfare, Government of India, 2020).

The awareness messages have asked people not to panic and strictly follow the given guidelines; the novel virus is infectious and is spreading widely, but the spread can be stopped or at least prevented if the safety measures are followed properly. Besides, the government has asked people to share information from verified sources so as to keep the sense of fear among the general public under control. In spite of all this, it was found that fear, panic, worry, and other mental health concerns have been on the rise among the general public (Bhat et al., 2020; Chakraborty and Chatterjee, 2020; Sood, 2020; Varshney et al., 2020). The findings of the current study suggest that those who are properly aware of the facts related to the COVID-19 pandemic and fall in the category of having excellent knowledge of COVID-19 were least anxious. A similar trend of findings has been highlighted by Roy et al. (2020), whereby it was reported that a moderate level of knowledge about coronavirus was related to a high level of anxiety among the respondents. There was adequate knowledge regarding the preventive aspects. People showed willingness to follow government guidelines on quarantine and social distancing. The other three groups have similar levels of anxiety overall and were found to be more anxious than the former. This is solely due to the fact that these groups of individuals lacked adequate scientific knowledge and understanding of the pandemic.

Following the trend reported above, with respect to mindfulness, it was found that the ones with excellent knowledge of COVID-19 had the highest level of mindfulness, followed by those with good and fair knowledge of COVID-19. The group having vague knowledge of COVID-19 was found to have the lowest level of mindfulness. The emergence of the virus has definitely been anxiety-provoking. One can have thoughts about what one could do to be better prepared and avoid adverse circumstances. Although most of the worry is related to the past or the future, proper knowledge and awareness regarding the pandemic helps in being calm and mindful of the present moment. Engaging mindfully in activities like hand washing, listening to others, doing household chores, or working from home helps to maintain composure even when faced with debilitating circumstances. Thus, the study rightly found that the higher the extent of knowledge of COVID-19, the greater the level of mindfulness.

Mindfulness can enhance cognitive flexibility as it is a component of the psychological flexibility construct. The findings show that the group having excellent knowledge of COVID-19 has the highest level of psychological flexibility, followed by those having good knowledge. The remaining two groups having fair and vague knowledge of COVID-19 have the lowest and similar levels of psychological flexibility. This means that individuals who are adequately informed about COVID-19 tend to be better aware and more accepting of their thoughts and emotions, walking on pathways leading to long-term benefits rather than that of immediate gratification and experiential avoidance. These factors enhance the state of being in contact with one’s values, overall well-being, and quality of life (Khandelwal, 2020; Zheng et al., 2020). All of these are found to be relatively less among those with good knowledge of the pandemic and least among those who are not well-informed about the same. The latter, besides being less aware or less accepting of their thoughts, might be engaging in maladaptive thoughts and actions as well as emotional avoidance, not being able to commit to doing what is valuable to the self (Baiano et al., 2020).

In general, the present sample uses positive coping strategies during these times. However, with respect to knowledge, the ones who had excellent knowledge of the pandemic are the ones who used putting into perspective an adaptive coping mechanism the most.

Correlational computation findings for correlation of knowledge of COVID-19 with the broad domains, namely, stress, anxiety, depression, overall mindfulness, and psychological flexibility, were significant at the 0.01 level (Table 3). Three cognitive emotion regulation strategies – other-blame, catastrophizing, and planning – were found to have a significant positive correlation with knowledge of COVID-19.

The detailed correlational analyses (Table 4 and Figures 2, 3) show that stress has a positive relationship with anxiety and depression while anxiety has a positive relationship with depression. Stress, depression, and anxiety are inversely related with mindfulness and its subdomains like acting with awareness and having a non-judging stance. Depression has an inverse correlation with the mindfulness subdomain of describing. Stress, depression, and anxiety have a positive relationship with other mindfulness subdomains like observing the internal and external world and non-reactivity and maladaptive cognitive emotion regulation strategies like self-blame, other-blame, rumination, and catastrophizing. Stress and anxiety have a positive relationship with positive refocusing, an adaptive cognitive emotion regulation strategy. Depression has a positive relationship with putting into perspective. Stress and depression have a positive relationship with acceptance. Finally, stress, depression, and anxiety have a negative correlation with psychological flexibility and knowledge of COVID-19.

Fear can be an adaptive response to perceived threat leading to fight-or-flight response. However, this response is supposed to help in dealing with the threat temporarily and not extend over months as is the case during the current COVID-19 pandemic. In case the fight-or-flight response becomes chronic, it paves way for stress, anxiety, depression, and suicidal tendencies. All of these are hence positively correlated with each other. The pandemic, the imposed restrictions, and its interference in daily life schedule are potential stressors that can affect both physical and psychological health and overall well-being. This might be due to the fact that the awareness that the infectious virus is around and can cause ill-health and fatal outcomes is taxing, thus leading to sadness and feelings of fear and worry (Cao et al., 2020; Li Z. et al., 2020; Rajkumar, 2020; Wang et al., 2020b; Özdin and Bayrak Özdin, 2020). As a reaction to this, depression and anxiety increase with the rise in stress level. This study was conducted in the second and third months of lockdown in India, and the trend of the findings justifies the psychological effects that the COVID-19 pandemic has on the community. Besides, anxiety too has a positive relationship with depression. Many researchers have also reported an increase in anxiety and depression owing to the ongoing pandemic (Alkwiese Sr et al., 2020; Holmes et al., 2020; Liang et al., 2020; Liu X. et al., 2020; Rajkumar, 2020; Roy et al., 2020; Sood, 2020; Varshney et al., 2020).

Researchers in India have reported an increase in anxiety and depression among Indians, owing to the pandemic (Goyal et al., 2020; Kumar and Nayar, 2020; Mishra et al., 2020; Rehman et al., 2020; Verma and Mishra, 2020). The current study shows that with the increase in anxiety symptoms like irrational fear, excessive worry, uncertainty regarding future, accompanying physiological reactions like palpitations, nausea, breathing difficulties, and repeated, ritualistic behavior comes an increase in depressive symptoms like apathy, low mood, concentration difficulties, agitation, anger, sleep and appetite disturbances, restlessness, fatigue, irritability, social withdrawal, ideas of helplessness, hopelessness, self-harm, and suicidal tendencies. These can be explained by the difficulties set in motion by the pandemic, like worry about health and safety, loss of jobs and financial problems, need to maintain proper safety measures, and mandatory social distancing norms (Robinson, 2020).

It is well evident from research that mindfulness can reduce stress, depression, and anxiety. It can calm an individual and has beneficial effects on one’s health and well-being by enhancing positive thinking, insight, adaptive coping strategies, and resilience (Endler and Parker, 1990; Fredrickson, 2001; Hofmann et al., 2010; Ramasubramanian, 2017). The current study findings suggest that the more one is aware of the self, delves deep in responding to the present moment with full awareness, accepts the way one is non-judgmental, and has unconditional empathy for self and others, the less stressful, depressed, and anxious one feels. Thus, adapting to the context set in motion by the pandemic can be easier by adopting the mindful stance (Zheng et al., 2020). The findings further suggest that the more one can talk about the experiences and observations during this time to self and others, label them adequately, and express oneself, the less depressed one is likely to feel.

The current findings also show that being more observant is related to higher levels of depression, anxiety, and stress among the participants. This might be due to the fact that dealing with COVID-19 requires us to be more cautious than usual. On one hand, from being alert of the precise duration of washing hands to wearing the protective equipment properly, the best way to deal with the “new normal” is to be observant of what is going on around us. However, being perpetually observant may take a toll on the individual. It can be a potential source of depression, anxiety, and stress considering the ever-increasing number of people getting infected followed by the trauma and panic reaction brought about by the same. On the other hand, seeing people not following adequate safety measures and maintaining safe distancing norms can be another stressor for some. Mental health issues are on the rise and being mindful of the ongoings of the psyche might be related to increased stress. Similar lines of factors also play a role behind the finding that an increase in seeking active detachment from negative thoughts and emotions and choosing not to react to them, however, brings about an increase in stress, depression, and anxiety overall. This probably implies that unlike the usual times, currently, the “defense” of being actively non-reactive is possibly bringing about more mental health issues among the participants.

Cognitive emotion regulation strategies have been known to have a significant relationship with emotional problems like depression and anxiety (Garnefski et al., 2002, 2004; Garnefski and Kraaij, 2006, 2007; Aldao et al., 2010; Aldao and Dixon-Gordon, 2014; Gross and Jazaieri, 2014; Liu and Thompson, 2017). The more one can make use of adaptive cognitive emotion regulation strategies, the better functioning state one’s psyche usually is in Legerstee et al. (2011). In the current study, it is found that an increase in depression, anxiety, and stress is linked with an increase in maladaptive cognitive emotion regulation strategies like self-blame, other-blame, rumination, and catastrophizing. Making use of maladaptive cognitive emotion regulation strategies in dealing with the adverse situations that arise out of the current context is indeed stressful (Restubog et al., 2020). However, this is not only relevant during this time of the pandemic, this has been noted in earlier literature as well (Bai et al., 2004). The current study shows that in order to deal with the disruptions caused by the COVID-19 pandemic, the more one is likely to blame self or others for the same, remain preoccupied with maladaptive thoughts, and focus on the worst possible outcome of events, the more likely it is for them to have an increase in their levels of depression, anxiety, and stress.

Moving onto the adaptive cognitive emotion regulation strategies, the findings suggest that an increase in stress and anxiety is related with an increase in positive refocusing. Depression has a positive relationship with the strategy of putting things into perspective. Further, an increase in stress and depression is linked with an increase in acceptance. These findings are a deviation from existing literature in this regard (Beck, 1999; Bouchard et al., 2004; Lee-Baggley et al., 2004), which may very well be due to the situations stirred up by the COVID-19 pandemic. Thinking and refocusing on positive and pleasant aspects, things, and situations have also become stressful currently, mostly owing to the uncertainty along with the restrictions imposed due to COVID-19. Hence, instead of reducing the mental health issues, it is working in the opposite direction by increasing stress and anxiety among the subjects. The more one tries to put things into perspective by thinking that many others have gone through worse experiences like dying or facing the death of family members/significant others owing to COVID-19, other comparatively worse things can happen in life than the ongoings, the more one gets entangled in depressive thoughts. This may be due to the fact that many known persons have been facing the fairly worse times of their life, making it difficult for one to focus one’s cognition in a positive direction. It might be that the more one tries to accept the situation one is in, with regard to the wide spread of the virus across the globe, the lockdown, and the inevitable change in daily basic lifestyle, the more helpless, stressful, and depressing it becomes for an individual.

Further, there is a negative correlation with psychological flexibility and knowledge of COVID-19 with depression, anxiety, and stress. In the current times, in order to adapt better to the “new normal,” it is essential to adapt to the fluctuations in the contextual demands, accept newer perspectives of living and surviving with the infectious virus around, and restructure the psychic resources so that a desirable balance can be achieved in the needs and demands at the moment. When one can achieve this, coping with the pandemic and its related aspects definitely gets easier, and as a result, the feeling of being overwhelmed, stressed, anxious, or depressed decreases. Conclusively, an increase in psychological flexibility brings about a decrease in depression, anxiety, and stress and vice versa (Kashdan and Rottenberg, 2010; Fledderus et al., 2013; Wersebe et al., 2018; Østergaard et al., 2020). With regard to the knowledge of COVID-19, it is obvious that the more aware an individual is of the current circumstances with respect to the virus, its nature, symptoms, safety measures, treatment options, and support provided by the government in dealing with the pandemic, the less likely it is for them to have mental health issues regarding the same.

In the domain of mindfulness, it was found that “observing” has a positive relationship with overall mindfulness and its subdomains of describing and non-reactivity, but a negative relationship with subdomains of acting with awareness and having a non-judging stance. In concurrence with the existing literature which shows that there is a positive interrelationship among all the five facets of mindfulness (Gu et al., 2016) as considered in this study, the current study shows similar trends; however, the exception has been in the case of the subdomains – acting with awareness and having a non-judging stance. This might be due to the fact that being continuously observant of the uncertainty and evolving nature of this pandemic situation may cause a certain degree of wear out, make one prone to be more pessimistic, and act with lesser awareness. A positive relationship has been found between “observing” and all the cognitive emotion regulation strategies. As the COVID-19 pandemic has introduced various layers of stressors in the daily lives of mankind, the more the individuals become observant of the same, the more they tend to make use of conscious, mental strategies depending on their usual repertoire of such strategies in order to deal with the challenges faced (Chambers et al., 2009; Dahl et al., 2015).

The “describing” subdomain of mindfulness has a positive relationship with overall mindfulness and its other subdomains of acting with awareness, having a non-judging stance and non-reactivity, all the adaptive cognitive emotion regulation strategies, psychological flexibility, and knowledge of COVID-19. It has an inverse relationship with catastrophizing, a maladaptive cognitive emotion regulation strategy. This suggests that the more an individual talks about his or her experiences related to the COVID-19 pandemic, the better are the chances of dealing adaptively with the stressors and accept the experiences without avoiding them. The more individuals can label what they feel and express it to themselves and others, the more they can face the situational demands adaptively. Being able to properly describe one’s experiences may be directly related to one’s level of self-awareness, being more in touch with the present moment and accept situations without being non-judgmental. This finding is substantiated by the works of Kabat-Zinn (1994) and Baer et al. (2006). Moreover, there is a lesser tendency among these individuals to think of irrational, catastrophic outcomes of events. This finding corroborates with the work of Su and Shum (2019).

The findings also show that the more an individual acts with awareness, the more is the use of non-judgmental stance, overall mindfulness, knowledge of COVID-19, and psychological flexibility. Acting with awareness has a negative correlation with non-reactivity, all negative cognitive emotion regulation strategies, positive refocusing, putting into perspective, and acceptance. Literature shows that the more one is mindful about one’s self and actions, the more is the ability to be present at the moment, be open to the experiences, and work with awareness (Kabat-Zinn, 1990a; Brown and Ryan, 2003; Shapiro et al., 2006; Thompson and Waltz, 2007; van den Hurk et al., 2011). In the context of the COVID-19 pandemic, the current study findings indicate that the more one acts with awareness, the better is their awareness of the pandemic itself, the understanding of the novel virus, its infectious nature, and the safety measures necessary to avoid transmission to self and others. The more one remains aware of the present moment, the more can one accept their experiences related to the pandemic and how it feels to respond adaptively to the experiences without avoiding them.

Further, the study also shows that the more an individual acts with awareness, the less is the use of maladaptive cognitive emotion regulation strategies like blaming self and others, catastrophizing, and ruminating excessively to cope with stressors. However, the study also shows that the more one is aware of the ongoings of the present moment in the context of the COVID-19 pandemic, i.e., the more one can focus attention on one’s current activities, the more is the possibility of one getting overwhelmed, stressed, and worried regarding the infectious spread of the virus, the unpleasant outcomes ensuing out of the pandemic condition, and the need to follow precautions that pose restrictions in one’s usual course of daily living. These are related to lesser tendency of an individual to focus on the positive and pleasant aspects of experiences after a negative outcome has taken place, shifting perspective from the maladaptive to the adaptive aspects and difficulty in accepting negative situations (Sood, 2020; Varshney et al., 2020). This is particularly related to the increase in depression and anxiety owing to the pandemic.

Being more non-judgmental is positively related with overall mindfulness and psychological flexibility and negatively related with all cognitive emotion regulation strategies. The correlation between mindfulness and psychological flexibility has been well-established by previous literature (Brown and Ryan, 2003; Fledderus et al., 2013; Wersebe et al., 2018). We assume that any kind of coping, be it adaptive/positive or maladaptive/negative by its inherent virtue, requires the effort to analyze and attach a meaning to it. This very process of taking cognizance of a situation and use a strategy to regulate emotions shall possibly defeat the idea of being non-judgmental to it. Hence, our findings indicate the same.

Being more non-reactive is positively related with overall mindfulness and all cognitive emotion regulation strategies. So, the more one can actively detach from negative thoughts and emotions and choose not to react to them, the more resilience, ability to be involved, and accepting the present moment’s experiences in a decentered manner can be expected. When this happens, the tendency to better use the cognitive emotion regulation strategies to cope up with stressors increases as well (Brown and Ryan, 2003; Chambers et al., 2009; Desrosiers et al., 2013; Finkelstein-Fox et al., 2018). When put in the context of the COVID-19 pandemic, these findings are similar to those delineated above.

Overall mindfulness has been to found to have an inverse relation with other-blame and catastrophizing but a direct correlation with all adaptive cognitive emotion regulation strategies, psychological flexibility, and knowledge of COVID-19. Hence, this suggests that being more mindful helps one to use more adaptive strategies to cope with the stressors posed by the COVID-19 pandemic, have better acceptance of experiences, and be more aware of the pandemic situation and the risks as well as challenges put forth during these times. All of these are related to reduction in stress, development of insight of the current situation, increase in positive thinking, resilience, and better coping mechanisms. The more there is increase in all these domains, the lesser is the tendency to use maladaptive cognitive emotion regulation strategies like incriminating others and having disastrous thoughts during such times. When the latter gets reduced, one can cope with the disturbances in daily living way better and well-being is increased both physically and psychologically (Feldman et al., 2007; Roemer et al., 2009; Jimenez et al., 2010; Garland et al., 2011; Finkelstein-Fox et al., 2018).

Psychological flexibility was found to be inversely related with all maladaptive cognitive emotion regulation strategies and two adaptive coping strategies, namely, putting into perspective and acceptance. It is understood from the existing literature in these domains that the more one can accept and adapt to fluctuating situational demands and balance one’s needs and desires with that of the contextual demands, the less one needs to make use of maladaptive cognitive emotion regulation strategies. However, there may be a threshold for the individuals to be accepting and adaptive. This may be the reason why the current study points to the fact that the former can be negatively related to shifting perspectives to adaptive aspects and acceptance. This can be particularly due to the inherent nature of the COVID-19 situation which has been showing us that no amount of preparation has been enough to deal with this pandemic, and when scientists have been struggling for months to come up with a concrete management plan, it is only justifiable that the general public may become less flexible in such conditions.

Further, knowledge of COVID-19 while being negatively related with other-blame and catastrophizing was found to be positively related with planning and psychological flexibility. This shows that remaining adequately updated with the latest information about COVID-19, its scientific understanding, precautionary measures, its effect on the health infrastructure, the society, and economic conditions of the nation is also related to lesser tendency toward blaming others for adverse experiences, expecting tragic outcomes and tendency to use better planning capacity to cope with the stressors while accepting the developments with openness and integrity. An interplay of these factors helps to ensure the well-being of an individual and the society collectively by making judicious use of existing medical infrastructure.

The third objective of the study was to determine whether knowledge of COVID-19, level of mindfulness, specific cognitive emotion regulation strategies, and psychological flexibility are significant predictors of depression, anxiety, and stress in the sample of the current study. Linear regression for 19 different variables was computed to predict anxiety level (Tables 5–7). These constituted eight demographic variables, namely, age, gender, no. of years of education, employment status, living in nuclear family, urban flag, no. of people living with, and chronic condition flag. Eleven other behavioral factors were considered for the analysis, which included specific cognitive emotion regulation strategy, level of mindfulness, and psychological flexibility.

Even though the ANOVA analyses showed no significant difference between gender categories, when controlled for other variables, gender was a significant predictor of anxiety, with males experiencing significantly a higher level of anxiety than females. This finding differs from previous studies which found females to be more prone to feeling anxious (Ebrahim et al., 2020; Liu N. et al., 2020; Özdin and Bayrak Özdin, 2020) and depressed (Özdin and Bayrak Özdin, 2020) during COVID-19.

In order to analyze depression, the same set of 19 different predictors was used (Tables 8, 9). People with a history of chronic health condition were more likely to have higher scores for depression. Given the fact that this group is reportedly more vulnerable during COVID-19 (Chen et al., 2020; Deng et al., 2020; Liu W. et al., 2020; Pan et al., 2020; World Health Organization, 2020), the findings are consistent with other similar studies (Rajkumar, 2020; Özdin and Bayrak Özdin, 2020). A higher level of psychological flexibility and overall mindfulness seemed to be inversely related with depression levels. Similar findings have been reported by other researchers (Bohlmeijer et al., 2010; Kashdan and Rottenberg, 2010; Wersebe et al., 2018; Zheng et al., 2020). Some emotional regulation strategies were significant markers of depression levels, like self-blame, other-blame, and acceptance (Farb and Segal, 2012).

The same set of 19 different predictors was used to analyze stress (Tables 10, 11). People with a history of chronic health condition were more likely to have higher scores for stress. The findings are akin to the ones obtained for depression and are in line with other researches (Bohlmeijer et al., 2010; Özdin and Bayrak Özdin, 2020). A higher level of psychological flexibility and overall mindfulness seemed to decrease stress level. The findings do not deviate much from previous literature in this context (Fledderus et al., 2013; Wersebe et al., 2018). Rumination was a significant marker of stress level. This is due to the fact that people tend to be more anxious if they keep ruminating on a particular thought, given the fact that we conducted this study at the time when the entire country was under lockdown (Satici et al., 2020).

Our findings also show that factors like gender, education level, and unemployment during these times were not significant predictors of either depression or stress levels. Unemployment and education were not significant predictors of anxiety. These findings are in concordance with another study which stated that the effect of COVID-19 on the general population’s work life had no effect on their psychological status and state anxiety (Liu X. et al., 2020).

## Conclusion

This study highlights the importance of being up-to-date with apt knowledge of COVID-19 from bona fide sources as it may be an important factor in dealing with anxiety, depression, and stress during a novel crisis. Additionally, the right amount of knowledge is also shown to have a direct relationship with being more mindful and cognitively flexible, both of which have been known to further decrease psychological distress. The findings also indicate that not all positive coping strategies (positive refocusing, for example) can help us to deal with our emotions in an adaptive manner and prolonged stress may result in being maladaptive. Our coping strategies thus need to be a balance between reality orientation and optimism. The two coping strategies that were found to be significant markers of anxiety and depression were self-blame and other-blame. The possibility of excessive time being spent on media these days may have been a potential contributor toward such coping strategies in our study as well. Since the uncertainty in dealing with this pandemic at a global level has been well-established, a very pertinent issue of relying on authentic sources of information cannot be stressed enough in order to collectively deal with the present times.

## Data Availability Statement

The raw data supporting the conclusions of this article will be made available by the authors, without undue reservation.

## Ethics Statement

Ethical review and approval was not required for the study on human participants in accordance with the local legislation. The participants provided their written informed consent to participate in this study.

## Author Contributions

ND is a Ph.D. scholar at the mentioned department, first, and corresponding author for this research, and involved conceptualization of the study, conducting survey of existing literature, data collection format development, contribution toward data collection, and manuscript writing and analysis. PP is a Ph.D. scholar at the mentioned department, second author for this research, and involved helping toward the conceptualization of work, conducting survey of literature, and contribution toward data collection and writing of the manuscript. DP is a Computer Science Engineer and pass-out of the Computer Science Department of the mentioned institute, and involved contribution toward data collection, statistical computations, data analysis, and writing the manuscript. All authors contributed to the article and approved the submitted version.

## Conflict of Interest

The authors declare that the research was conducted in the absence of any commercial or financial relationships that could be construed as a potential conflict of interest.
